# Severe H7N9 Infection Is Associated with Decreased Antigen-Presenting Capacity of CD14^+^ Cells

**DOI:** 10.1371/journal.pone.0092823

**Published:** 2014-03-24

**Authors:** Hongyan Diao, Guangying Cui, Yingfeng Wei, Jianing Chen, Jian Zuo, Hongcui Cao, Yu Chen, Hangping Yao, Zhigang Tian, Lanjuan Li

**Affiliations:** 1 State Key Laboratory for Diagnosis and Treatment of Infectious Diseases, The First Affiliated Hospital, School of Medicine, Zhejiang University, Hangzhou, Zhejiang, China; 2 Collaborative Innovation Center for Diagnosis and Treatment of Infectious Diseases, Hangzhou, China; 3 Institute of Immunology, Medical Center, University of Science & Technology of China, Hefei, China; University of Rochester, United States of America

## Abstract

The outbreak of H7N9 human infection has caused concern worldwide, but the immunological characteristics of infected patients and the determinants of diverse outcomes remain to be thoroughly understood. In this study, twenty-three patients with H7N9 infections were classified into severe and mild cases. We found that severe patients were commonly lymphopenic with significantly lower levels of T cells, monocytes and related cytokine levels compared to the mild cases. The expression of HLA-DR on CD14^+^ cells were significantly lower in the severe infection group compared to the mild group (in acute phase: 34.65±4.88 vs. 10.37±1.69, *p*<0.001). Importantly, the expression of HLA-DR on CD14^+^ cells was negatively correlated with H7N9 infection severity. Furthermore, although the phagocytosis capabilities of monocyte were similar between two groups, the monocytes of severe infection patients had a lower antigen-presenting capacity. And some in vitro experiments suggested that the impaired antigen-presenting function is associated with lower activation of T cells in responses to immune stimulation. Our present study suggested that the severe H7N9 patients were in a state of immune decrease which presented with general lymphopenia and low antigen-presenting capacity resulting in impaired T cell response. Additionally, HLA-DR levels of CD14^+^ cells may be a potential biomarker for predicting H7N9 disease progression.

## Introduction

The avian influenza A (H7N9) virus was first reported to cause human infection in eastern China in 2013. The cases occurred in a first wave (n = 133) from February through May 2013 and decreased during the summer. However, since October 2013 a second wave of H7N9 human infection has been occurring [1]. With the exception of those caused by the H5N1 [2] and H7N7 [3] subtypes, Avian influenza virus (AIVs) infections were generally presented with mild manifestations such as conjunctivitis and upper-respiratory-tract infections [4]. But the confirmed H7N9 cases of human infection were characterized by very severe disease or even fatal outcomes [5].

The virus and host immune status are primary factors which influence the consequences of virus infection. These factors are not mutually exclusive, and it is conceivable that both contribute to the complex pathogenesis of viruses. Two recent studies suggested that H7N9 AIVs possess some mutations which are associated with mammalian adaptation or increased virulence [4], [6]. Several studies investigated the biological features of H7N9 patients has been report. Zhou et al. revealed the increased levels of the chemokines and cytokines in acute serum samples [7]. Wang et al. found that early hypercytokinemia in H7N9 patients is associated with interferon-induced transmembrane protein-3 dysfunction and predictive of fatal H7N9 infection [8]. Chen et al. found that the levels of T cell subsets were lower in critically ill H7N9 patients than in recovered patients [9]. However, to date, the immune status of H7N9 patients has not been well understood. In H5N1 infection and the severe H1N1 pandemic of 2009, lymphocytopenia was significantly associated with the disease severity [10], [11]. CD8^+^ T cells, operating either via direct lysis of infected cells or by the production of pro-inflammatory cytokines such as IFN-γ, are critical for the efficient resolution of influenza virus infections in animal models.

Studies revealed that the downregulation of major histocompatibility complex (MHC) class II (HLA-DR) antigens on monocytes/macrophages was involved in an immune depression status [12]. Studies of human immune responses *in vitro have shown that* T cell responses to all influenza HA molecules are restricted by HLA-DR molecules [13]. Reduced expression of HLA-DR molecules on monocytes has been observed in patients that are critically ill as a result of liver failure, septic shock and burn injury [14], [15], [16]. Furthermore, some studies have revealed that this alteration is associated with reduced antigen-presentation capacity and poor outcomes [17].

Although H7N9 patients generally presented with relatively severe illness, some patients recover, while disease progression and even death occurs in other cases [5], [18]. However, the determinants of such diverse outcomes of H7N9 infections are not well understood.

In this study, we investigated the immune status of patients with confirmed H7N9 infection to clarify the immunological differences between mild and severe H7N9 patients. In contrast to the mild cases, we found that severe patients were in a state of immune decrease which manifested as lymphopenia, low antigen-presenting capacity and reduced inflammatory responses. Moreover, we found that HLA-DR levels on CD14^+^ monocytes were significantly lower in severe cases. The expression level of HLA-DR on CD14^+^ monocytes was negatively correlated with the severity of the H7N9 infection. These data suggested that decreased expression of HLA-DR on CD14^+^ monocytes is a potential indicator of the severity of H7N9 infection.

## Materials and Methods

### Patients and associated procedures

Our study was conducted in 23 hospitalized patients with laboratory-confirmed H7N9 virus infection recruited at the First Affiliated Hospital College of Medicine, Zhejiang University. This study was approved by the institutional review board of the First Affiliated Hospital College of Medicine, Zhejiang University (reference number 2013-166). The requirement for informed consent was waived because of the urgent need to collect data on this emerging pathogen. All the patients presented with new-onset respiratory symptoms and unexplained radiographic infiltrate [4]. To investigate the immune status in patients with different disease severity, we divided the subjects into mild patients (n = 13) and severe patients (n = 10). Briefly, subjects were categorized as severe patients if they met one or more of the following criteria (during the whole course of hospitalization): (i) respiratory failure, (ii) respiratory distress syndrome (ARDS), (iii) multiple organ dysfunction syndrome (MODS), (iv) received advanced respiratory support: non-invasive ventilation by continuous positive airway pressure/mechanical ventilation/extracorporeal membrane oxygenation (ECMO). Clinical data, including disease history, physical examination and hematological investigation results were collected and analyzed for all enrolled patients. The Pandemic Medical Early Warning Score (PMEWS) [19] and acute physiology and chronic health evaluation II (APACHE-II) score [20] of each enrolled subject was calculated as a measurement of disease severity. The day of clinical symptom onset was assigned as day 0. The acute phase was defined as day 0 to day 10 from clinical symptom onset, while the recovery phase was defined as day 11 to day 27.

### Evaluation of patient H7N9 viral load

All laboratory procedures on respiratory secretions were carried out as previously described [4]. H7N9 infection was confirmed by RT-PCR._ENREF_26_ENREF_18 And the cycle threshold (Ct) values of FLUA, H7 and N9 in endotracheal aspirates of patients were used to evaluate the viral load.

### Flow cytometry

The following monoclonal antibodies were used in the present study: FITC-CD4/PE-CD8/PerCP-CD3, FITC-CD3/PE-anti-CD 16+56, FITC-CD19, and PECy5-CD14 (clone RMO52) were purchased from Beckman Coulter, CA, USA. FITC-HLA-DR (clone Immu-357) was purchased from BD Pharmingen, CA, USA. PE-BDCA-1, PE-BDCA-2, and PE-BDCA-3 were purchased from Miltenyi, Germany. Intracellular staining for PE- IL-17 (clone eBio64CAP17, eBioscience, USA) and PE-IFN-γ (clone 4SB3, eBioscience, USA) were executed with Cytofix/CytopermFixation/Permeabilization Kit (BD Pharmingen, CA, USA) according to the manufacturer's instructions. Flow cytometry was performed on the Beckman Coulter FC 500 MPL with CXP software.

### Isolation and culture of PBMC

In the time period of 7-9 days after clinical symptom onset, PBMCs were isolated by density centrifugation on Ficoll according to the manufacturer's instructions. Cells were then cultured at 2×10^6^ cells/ml in 10% FCS-RPMI-1640 containing polyI:C (20 μg/ml, Sigma-Aldrich, St. Louis, MO, USA) for 48 h and heat-inactivated H7N9 virus (isolated from hospitalized patients at the First Affiliated Hospital College of Medicine, Zhejiang University, 10 μg/ml) for 8 h.

### Phagocytosis assay

PBMCs (2×10^6^ cells/ml) were incubated with red fluorescent protein (RFP)-labeled *E. coli* (2×10^7^/ml) for 50 min. Subsequently, cells were incubated with FITC-HLA-DR and PECy5-CD14 antibodies according to standard procedures. The percentage of positive stained cells was analyzed by flow cytometry. Phagocytic internalization of DAPI-labeled *E. coli* was confirmed by fluorescence microscopy.

### Antigen presentation function of CD14^+^ cells

Mouse Anti-Human CD14 and CD4 Microbeads were used to isolate CD14^+^ cells and CD4^+^ cells from PBMCs of patients with severe or mild H7N9 infection as well as health controls by Magnetic activated cell sorting (Miltenyi Biotec. Germany). Then patients' CD14^+^ cells stimulated with polyI:C (20 ng/ml) were co-cultured with CD4^+^ cells from health control for 18 h. Intracellular IFN-γ expression in CD4^+^ T cells from health control were executed with Cytofix/CytopermFixation/Permeabilization Kit. Meanwhile, CD14^+^ cells from health control were co-cultured with CD4^+^ cells from patients and the IFN-γ expression in CD4^+^ T cells were detected.

### Cytokine and chemokine analysis

Host immunological responses were evaluated with the Luminex enzyme immunoassay (Luminex, TX, USA) according to the manufacturer's protocol. The levels of 27 cytokines and chemokines were analyzed in vivo and in vitro (Bio-Plex Pro human cytokine group I, USA)

### ELISPOT assay

According to the manufacturer's instructions, IFN-γ and IL-17 ELISPOT assays were performed using commercially available kits (eBiosciences, USA). PBMCs were cultured with polyI:C (20 ng/ml) for 24 h, and the positive cells enumerated by an ImmunoSpot S5 Macro Analyzer (C.T.L., Shaker Heights, OH, USA) were expressed as numbers of IFN-γ or IL-17 spot-forming units per well.

### Statistical analysis

Continuous variables were calculated as means ±SD. For categorical variables, the percentages of patients in each category were calculated. An unpaired Student's *t*-test or Fisher's exact test, as appropriate, was used for statistical analysis of comparisons between the mild and severe patients. Pearson's correlation analysis was performed for normally distributed variables and Spearman's rank correlation analysis, for non-parametric variables. Data are representative of at least two independent experiments. A *P*-value of less than 0.05 was considered to indicate statistical significance. *, *p*<0.05. **, *p*<0.01, ***, *p*<0.001. All analyses were performed using SPSS software.

## Results

### Clinical features of mild and severe H7N9 patients

**Figure 1:**
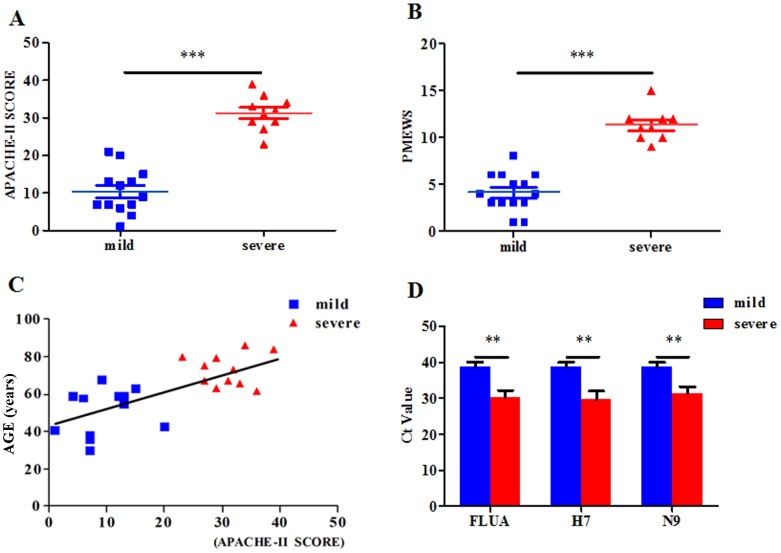
The APACHE-II and PMEWS scores and Ct value in patients. High APACHE-II scores (A) and the high PMEWS scores (B) were observed in severe patients compared with mild patients; (C) The ages of patients versus APACHE-II score with Spearman's correlation coefficients. Each point represents an individual patient. Results are shown for patients with severe H7N9 infection (red symbols) with mild H7N9 infection (blue symbols). (D) The Ct values on the 10th day after symptom onset in mild and severe patients. Data represent mean ±SD.

Our study was conducted in 23 (male  = 12, female  = 11) hospitalized and confirmed H7N9 patients, enrolled from 18 April to 20 May, 2013. The clinical characteristics of the enrolled patients are summarized in Table 1. Severe and mild patients showed no significantly difference in terms of sex, temperature, smoking history, glucocorticoid given, and underlying medical disorders. But, the incidence of dyspnea was notably higher in the severe group (10/10) compared with the mild group (0/13) (Table 1). Furthermore, significantly higher PMEWS scores (11.3±1.64 vs. 4.5±2) and APACHE-II scores (31.3±4.60 vs. 10.38±5.73) were observed in the severe group compared with mild group (Fig. 1A & B), thus, proved the validity of the patients' classification. The ages of the patients in the severe group were significantly higher (72.7±8.92 years) than those in the mild group (52.6±13.63 years). Moreover, a positive correlation was observed between the severity of H7N9 infection (APACHE-II score) and patients' age (Fig. 1C). In addition, we compared the RT-PCR cycle threshold (Ct) values of FLUA, H7 and N9 in endotracheal aspirates of mild and severe H7N9 patients as the procedures described in our previous study [4]. Interestingly, the H7N9 viral load was higher and the duration of virus infection was longer in the severe group than that in the mild group (Fig. 1D, Table 1). These data suggested that the level of viral load and persistence might influence the outcome of infection.

**Table 1:** Epidemiological and clinical features of patients with H7N9 virus infection.

	Mild	Severe	p-value
Age	52.6±13.63 (30–75)	72.7±8.92 (62–86)	<0.001
Sex(M/F)	8/13 (61.5)	4/10 (40)	0.327
Underlying medical disorders	7/13 (53.8)	9/10 (90)	0.066
Chronic smoker	3/13 (23.1)	0/10 (0)	0.113
Days hospitalized (deaths removed)	19.8±5.17 (12–28)	33.3±5.99 (20–40)	<0.001
Conjunctivitis	0/13 (0)	1/10 (10)	0.264
Cough	12/13 (92.3)	7/10 (70)	0.177
Sputum	11/13 (84.6)	6/10 (60)	0.199
Hemoptysis	2/13 (15.3)	0/10 (0)	0.212
Dyspnea	1/13 (7.6)	10/10 (100)	<0.001
Nausea or vomiting	1/13 (7.6)	1/10 (10)	0.854
Diarrhea	0/13 (0)	1/10 (10)	0.264
Myalgia	1/13 (7.6)	2/10 (20)	0.408
Fatigue	8/13 (61.5)	2/10 (20)	0.049
Adapted PMEWS^a^	4.5±2 (1–8)	11.3±1.64 (9–15)	<0.001
APACHE-II^b^	10.38±5.73 (1–20)	31.3±4.60 (23–39)	<0.001
Time between onset of symptoms and initiation of oseltamivir (days)	6±4.5(3–20)	9±3.8 (4–15)	0.146
Time between onset of symptoms and negative conversion of H7N9 virus (days)	7±1.53 (5–9)	14.75±2.71 (10–19)	<0.001
Glucocorticoid given	4/13 (30.7)	3/10 (30)	0.97
ARDS	0/13 (0)	7/10 (70)	0.723
Shock	0/13 (0)	3/10 (30)	0.035
Respiratory failure	0/13 (0)	10/10 (100)	<0.001
Disturbance of consciousness	0/13 (0)	0/10 (0)	
MODS	0/13 (0)	6/10 (60)	<0.001
Advanced respiratory support^c^	0/13 (0)	9/10 (90.0)	<0.001

a Adapted PMEWS  =  Adapted Pandemic Medical Early Warning Score.

b APACHE-II  =  Acute physiology and chronic health evaluation II.

c Advanced respiratory support included non-invasive ventilation by continuous positive airway pressure, mechanical ventilation and extracorporeal membrane oxygenation. Categorical variable data are given as positive/tested (%). Continuous variable data are given as mean±SD (range).

### Leucocyte subset counts and their correlation with H7N9 infection severity

**Figure 2:**
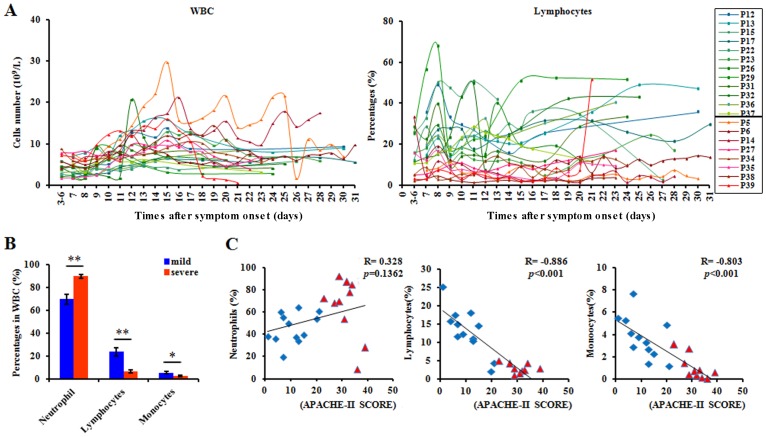
Leucocyte subset counts and their correlation with the severity of H7N9 infection. (A) The total number of leucocytes and the percentages of lymphocytes in patients during disease progression. Each line represents an individual patient. Results are shown for patients with severe H7N9 infection (red tones symbols and lines) with mild H7N9 infection (blue tone symbols and lines). (B) The percentages of neutrophils, lymphocytes and monocytes in patients on the 7th day after symptom onset. Data represent mean ±SD. (C) the lowest percentages for neutrophils, lymphocytes and monocytes vs. APACHE-II score with Spearman's rank correlation coefficients. Each point represents an individual patient. Results are shown for patients with severe H7N9 infection (red tones symbols and lines) with mild H7N9 infection (blue tone symbols and lines).

Most of the individuals presented leucopenia (<4×10^9^/L) and lymphocytopenia in the early stages after symptom onset (Fig. 2A & Fig. S1A). The percentages of lymphocytes and monocytes decreased in the severe patients compared with the mild patients during the disease progression (Fig. 2A & Fig. S1B). The percentages of neutrophils increased slightly in the severe group, but there was no significant difference in the absolute neutrophil counts between the mild and severe groups (Fig. S1C & D). Importantly, a negative correlation was observed between the severity of H7N9 infection (APACHE-II score) and the percentages of lymphocytes and monocytes, but no obvious correlations was found between APACHE-II score and the http://www.iciba.com/neutrophile_granulocyteneutrophil percentage (Fig. 2C). These date suggested that the severity of H7N9 infection was closely related to the count and percentage of lymphocytes.

### Cytokine and chemokine analysis of peripheral blood of H7N9 patients

**Figure 3:**
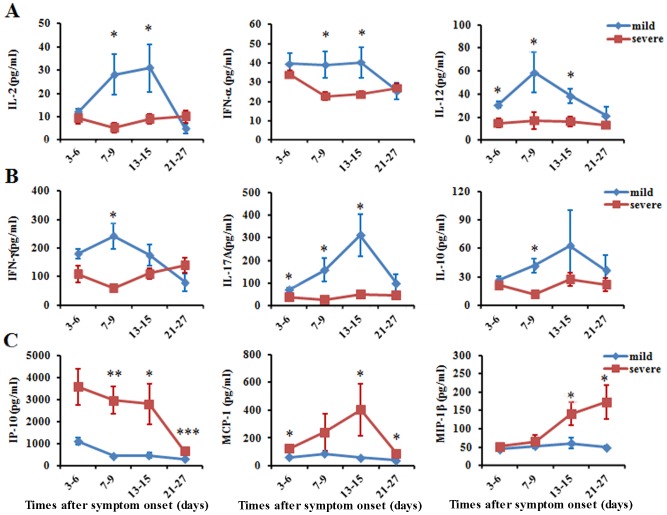
Levels of plasma cytokines and chemokines over the course of H7N9 infection. (A) Plasma levels of IFN-α, IL-2 and IL-12; (B) IFN-γ, IL-17A and IL-10; (C) IP-10, MCP-1 and MIP-1β in patients with severe versus mild H7N9 infection on different days after the onset of symptoms. Data represent mean ±SD for patients with severe H7N9 infection (red tones symbols and lines) and with mild H7N9 infection (blue tone symbols and lines). Experiments were performed in duplicates and repeated at least three times.

Strong inflammatory responses characterized by significantly elevated circulating cytokines and chemokines have been observed in H5N1 virus infections [21]. According to our study, in the mild group, most cytokines increased in the acute stage and then decreased in the recovery phase, while these cytokines were consistently detected at lower levels in the severe group during the disease progression (Fig. 3). The levels of cytokines produced by antigen-presenting cells (APCs), including IL-12 and IFN-α, were significantly lower in the severe group compared with the mild group (Fig. 3A). Compared to severe group, Th1 cytokines (IFN-γ and TNF-α), Th2 cytokines (IL-10) and IL-17A were significantly increased in the acute phase of disease in the mild group (Fig. 3B & Fig. S2). The levels of other cytokines, including IL-6, IL-4, IL-1β and IL-5, exhibited a similar trend (). However, levels of chemokines (IP-10, MIP and MCP-1) that are mostly produced in bronchial epithelial cells and alveolar macrophages [22], [23] were higher during the disease progression in the severe group compared with the mild group, may indicate severe lung injury in the severe group(Fig. 3C). These data suggested that some of the cytokines were reduced in severe H7N9 patients, while several chemokines which involved in the lung injury were increased in severe patients.

### Characteristics of lymphocyte subsets in peripheral blood

**Figure 4:**
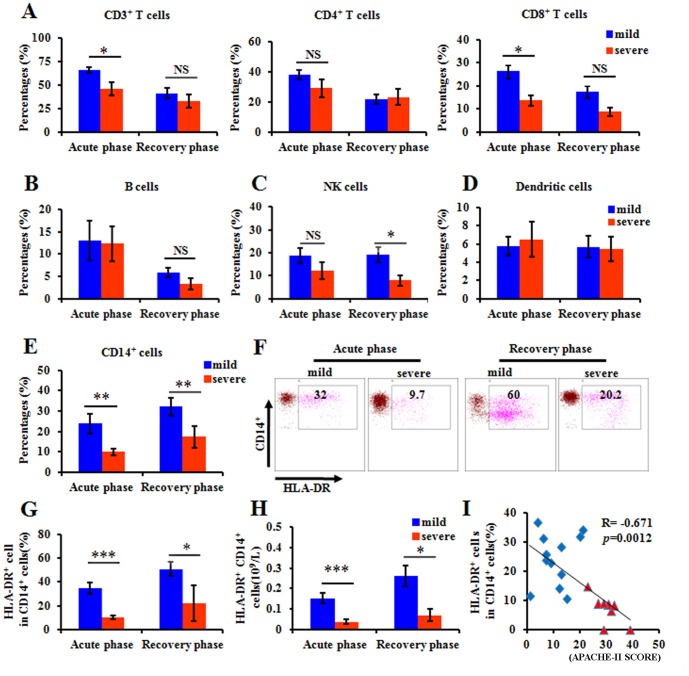
The percentages of lymphocyte subsets in peripheral blood. The distribution of CD3^+^ cells, CD4^+^ cells, CD8^+^ cells(A), B cells (B), NK cells (C), dendritic cells (D), CD14^+^ cells (E) and the percentages of HLA-DR expression on CD14^+^ monocytes (F and G) in patients during acute and recovery phase were detected by flowcytometry. Data represent mean ±SD. (H) The absolute numbers of HLA-DR^+^CD14^+^ monocytes in patients during acute and recovery phase were detected by flowcytometry. Data represent mean ±SD. (I) The lowest percentages for HLA-DR expression level on CD14^+^ monocytes versus APACHE-II score with Spearman's correlation coefficients. Each point represents an individual patient. Results are shown for patients with severe H7N9 infection (red symbols) with mild H7N9 infection (blue symbols).

Analysis of peripheral blood cells revealed obvious lymphocytopenia in the severe patients compared with the mild patients. We then examined alterations in the lymphocyte subsets in the patients with H7N9 infection. In the acute phase, the percentages of CD3^+^ and CD8^+^ T cells were significantly lower in the severe group than those in the mild group, although these differences disappeared gradually during the recovery stage (Fig. 4A). No remarkable difference was noted in the percentages of CD4^+^ T cells, B cells (CD19^+^) and dendritic cells between the two groups with H7N9 infection (Fig. 4A-D). We detected no significant difference on NK cells during the acute phase, however, the percentage of NK cells were decreased during recovery phase in severe H7N9 patients (Fig. 4C). Importantly, the percentages of CD14^+^ cells were significantly decreased in the severe group in both the acute and recovery phases (Fig. 4E).

HLA-DR plays a critical role in antigen-presentation during pathogen infection. To further investigate the function of CD14^+^ cells, which is constituted mainly by monocyte, we detected the expression of HLA-DR on these CD14^+^ cells. In the patients with severe H7N9 infection, the percentages and absolute counts of CD14^+^ monocytes expressing HLA-DR were significantly lower than those in the subjects in the mild group during the course of disease progression (Fig. 4 F–H). Importantly, correlation analysis showed that the level of HLA-DR expressing on CD14^+^ monocytes was negatively related to the severity of H7N9 infection disease (Fig. 4I). However, table 1 showed that there was significant age difference between mild and severe cases. To take the age factor into account, we enrolled two group health subjects whose age was similar to the mean age of mild and severe H7N9 patients, respectively. And their characteristics of lymphocyte subsets in peripheral blood were detected by flow cytometer. The results showed that, between these two groups, there were no significant differences on the percentages of CD14^+^ cell, HLA-DR expression in CD14^+^ cells and other lymphocyte subsets (Fig. S3). Decreased CD14^+^ cell rate and the down-regulated expression of HLA-DR on CD14^+^ cells implied that the antigen-presenting capacity might be reduced in severe patients.

### Decreased antigen-presenting function of CD14^+^ cells in patients with severe H7N9 infection

**Figure 5:**
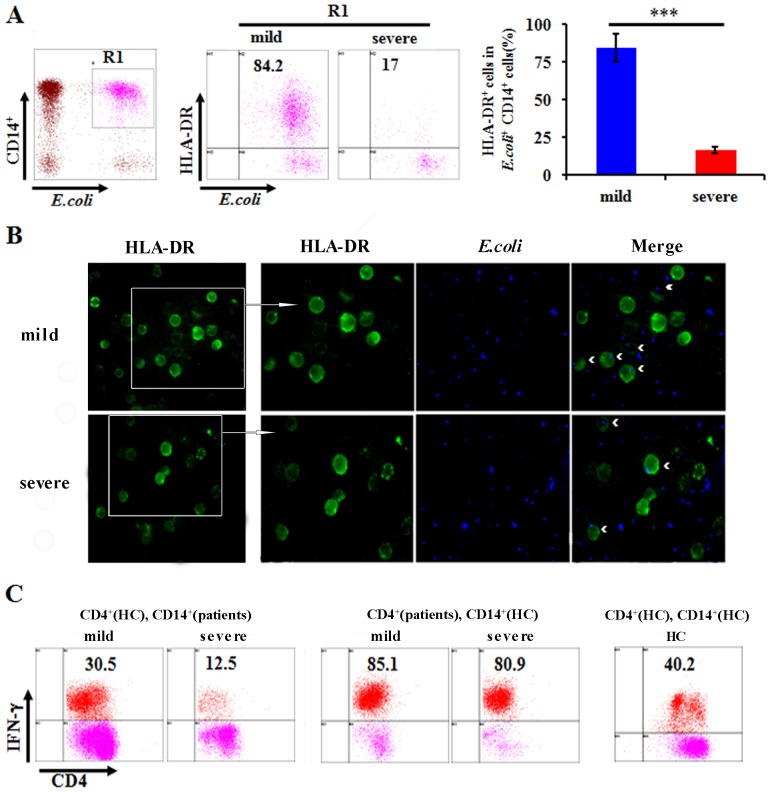
Phagocytosis and antigen presentation capacity of CD14^+^ cells. (A) Percentages of HLA-DR expressing on *E.coli* ^+^ CD14^+^ cells following RFP-labeled *E. coli* stimulation. Data represent mean ±SD. (B) HLA-DR (stained by green) expressions on phagocytic internalization of *E. coli* stained by DAPI (blue) in PBMC (shown by red arrows in merged image) was confirmed by fluorescence microscopy. Original magnification: ×400 & ×600. (C) Intracellular IFN-γ expression in CD4^+^ T cells from health control (HC) after co-cultured with CD14^+^ cells from patients with severe and mild H7N9 infection (the ratio of CD14^+^ cells/CD4^+^ T cells is 1:1) following polyI:C stimulation (20 ng/ml) (left); intracellular IFN-γ expression in CD4^+^ T cells from patients with severe and mild H7N9 infection after co-cultured with CD14^+^ cells from HC following polyI:C stimulation middle); intracellular IFN-γ expression in CD4^+^ T cells from HC after co-cultured with CD14^+^ cells from HC following polyI:C stimulation (right).

Phagocytosis of microbes and antigen presentation are the fundamental functions of monocytes. Evaluation of phagocytosis by internalization of *E. coli* showed that there was no difference in the phagocytic capacity of PBMC in the severe and mild group (Fig. S4). However, the percentage of HLA-DR^+^ cells on the CD14^+^ *E.coli* ^+^ cells that had phagocytized RFP-labeled *E. coli* was significantly lower in the severe group compared with mild group (Fig. 5A). Meanwhile, Phagocytic internalization of *E. coli* was confirmed by fluorescence microscopy and the similar results in PBMC were observed (Fig. 5B). To further investigate whether the antigen-presenting function of CD14^+^ monocytes was reduced in the severe group, we adopted an *in vitro* system in which polyI:C stimulated CD14^+^ cells were co-cultured with CD4^+^ T cells. We isolated CD14^+^ and CD4^+^ T cells from the severe and mild H7N9 patients as well as healthy control individuals. IFN-γ production in CD4^+^ T was determined by flow cytometry. When CD4^+^ T cells from healthy controls were co-culture with CD14^+^ cells from severe and mild H7N9 patients, we found that IFN-γ production in CD4^+^ T cells were significantly decreased in severe H7N9 group compared with the mild group (Fig. 5C). In addition, no significant differences were detected in IFN-γ production of CD4^+^ T, when CD14^+^ cells from healthy controls were co-culture with CD4^+^ T cells from severe and mild H7N9 patients (Fig. 5C). These data suggested that the antigen-presenting function of CD14^+^ monocytes was remarkably attenuated in the severe H7N9 group without decreased phagocytosis.

### Reduced production of cytokines and chemokines by T cells in vitro

**Figure 6:**
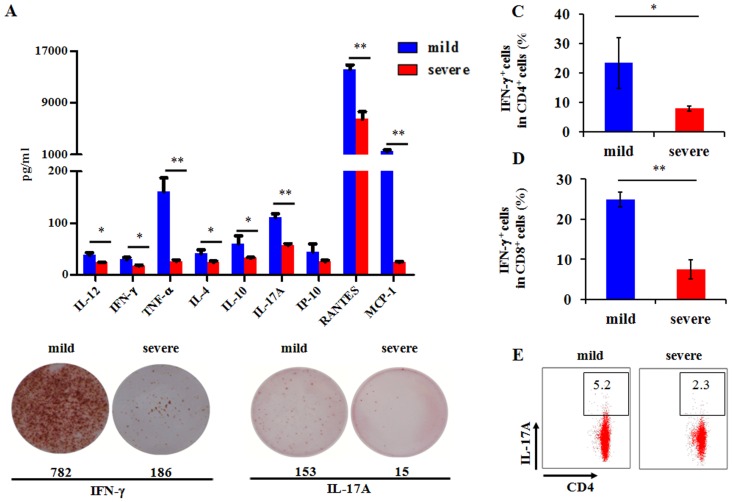
In vitro analysis of cytokine and chemokine production by patients PBMC. (A) The levels of cytokines and chemokines secreted by inactivated H7N9 virus (1Moi) stimulated PBMC from patients with severe and mild H7N9 infection (5 μl/ml). Data represent mean ±SD. (B) ELISPOT analysis of IFN-γ and IL-17 secreting cells following polyI:C stimulation of PBMC from patients with severe and mild H7N9 infection (20 ng/ml). All ELISPOT assays were performed in duplicate. Intracellular IFN-γ expression in CD8^+^ T (C), CD4^+^T (D) cells and intracellular IL-17A expression in CD4^+^T (E) cells after heat inactivated H7N9 virus stimulation was analyzed by flow cytometry. Data represent mean ± SD.

The ability of PBMCs to secrete cytokines and chemokines in response to inactivated H7N9 virus or polyI:C (a dsRNA analog) stimulation was measured in vitro. The concentrations of six cytokines (IL-12p70, IFN-γ, TNF-α, IL-4, IL-10 and IL-17A) and two chemokines (MCP-1 and RANTES) were significantly decreased in the severe group compared with the mild group upon stimulation of inactivated H7N9 virus (Fig. 6A) or polyI:C (Fig. S5). Furthermore, ELISPOT assays showed that the numbers of IFN-γ and IL-17A secreting cells were significantly decreased in the severe group (Fig. 6B). In addition, IFN-γ production in CD4^+^ T and CD8^+^ T cells as well as IL-17 in CD4^+^ T cells were significantly decreased in the severe group compared with the mild group after heat-inactivated H7N9 virus stimulation analyzed by flow cytometry (Fig. 6C–E). These data obtained from in vitro experiments were consistent with the results of cytokine levels in peripheral blood of H7N9 patients, suggested that the T cell response to H7N9 infection was reduced in severe H7N9 patients.

## Discussion

The novel influenza A subtype H7N9 virus was first found to cause human infection in eastern China_ENREF_28. Although the H7N9 infected patients generally presented with severe symptoms, some patients recovered from this disease, while others experienced steady deterioration or even death [5], [18]. However, the underlying mechanisms leading to different outcome of H7N9 infection remain unclear. To clarify the factors affecting the outcome of H7N9 infection, we limited the focus of our study to the immune status of confirmed H7N9 patients. The enrolled patients were divided into mild and severe groups and there were no significant differences in the clinical characteristics, such as sex, underlying medical disorders and smoking history between the patients of different group. However, the mean age in the severe group was significantly higher than that in the mild group and a positive correlation was observed between the severity of H7N9 infection and patients' age, suggesting that age is a risk factor in determining the outcome of H7N9 infection. The very young and the very old are the most susceptible to emerging viral diseases due to immunological naive and immune-senescence, respectively [24]. The median age of the H7N9 patients was 61 years, therefore, we speculate that potentially decreased immune response in old patient may influence the severity of infection.

Analysis of the changes in the peripheral blood leucocyte subgroups in H7N9 patients revealed that lymphopenia was observed in most patients, particularly in patients with severe illness. The lymphopenia observed here was similar to that reported in AIV H5N1 [10], [25] and severe pandemic H1N1 2009 infections [11]. Moreover, the percentages of CD3^+^ T cells were significantly lower in the severe group, while no differences in B cells and dendritic cells were detected. However, we detected a decrease in CD14^+^ cells, which is constituted mainly by monocyte, in the severe group compared with the mild group.

Monocytes perform phagocytosis and antigen presentation functions. Studies have shown decreased HLA-DR expression of monocyte, an important molecule involved in antigen-presentation, in severe sepsis [26]. We found that the percentages of CD14^+^ cells were significantly lower in the severe group than in the mild group during the early and late phases of disease. Furthermore, HLA-DR expression on CD14^+^ cells was significantly reduced in the severe group, with a significant negative correlation observed between the level of HLA-DR^+^CD14^+^ cells and the severity (APACHE-II score) of H7N9 infection. Previous studies revealed that glucocorticoid given can alter CD14 expression and HLA-DR expression in immune cells. But in this study, there was no significant difference in glucocorticoid given between mild and severe H7N9 patients (*p* = 0.97). Thus, the down-regulation of the HLA-DR^+^CD14^+^ cells was related to the severity of H7N9 infection. Dendritic cells are also potent APCs, however, there was no significant difference in the expression of the DC markers BDCA1, BDCA2 and BDCA3 between the two groups. No differences were observed in the phagocytic capacity of PBMC from the mild and severe groups; however, the expression of HLA-DR^+^ *E.coli* ^+^ CD14^+^ cells was obviously decreased in the severe group compared with that in the mild group, and we further found that IFN-γ production in CD4^+^ T cells were significantly decreased after co-cultured with CD14^+^ cells from the severe H7N9 patients compared with the mild patients. These data suggested marked attenuation of the antigen-presenting function in severe patient. Antigen-presenting cells can induce T cell responses. Thus, we speculated that the weak T cell response observed in the severe group was related to the function of HLA-DR on CD14^+^ cells. Compared to severe group, Th1 cytokines (IFN-γ and TNF-α), Th2 cytokines (IL-10) and IL-17A were significantly increased in the acute phase of disease in the mild group. The similar results were obtained by using cytokine analysis of PBMC following inactivated H7N9 virus stimulation in vitro. These data suggested that T cell responses were attenuated in severe H7N9 patients. However, the concentrations of the plasma chemokines, IP-10, MCP-1 and MIP-1β, were generally higher in the severe group. Previous studies had revealed that these chemokines were produced by bronchial epithelial cells or alveolar macrophages_ENREF_33 and were elevated in more severe H5N1 patients [23]. We speculated that these elevated chemokines might be an indicator of serious lung injury rather than a direct consequence of the viral infection. Collectively, severe H7N9 patients commonly exhibited lymphopenia, low antigen-presenting capacity and weak T cell responses. It could be speculated that this state of immune decrease lengthened the duration of viral persistence in severe patients due to a compromised capacity to eliminate virus.

Similar to the H7N9 virus, the H5N1 virus tends to cause serious disease in humans [27]. But Jong et al. showed that severe H5N1 patients presented with higher levels of cytokine secretion than mild patients [23]. The median age of the H7N9 patients was 61 years (range, 3–88), with 42.3% of the patients aged 65 years or older [28]. However, the age distribution of hospitalized H5N1 patients was significantly different. The median age of 26 confirmed H5N1 patients was 29 years (range, 6–62) [29]_ENREF_19. Immune-senescence occurs with age increasing [24], thus, old age may be one factor of reduced immune responses. In addition, our study revealed that severe H7N9 patients possessed attenuated antigen-presenting capacity compared to mild patients which can lead to reduced cytokine secretion. Consistent with our data, it was previously shown that IL-17, IL-2, IL-4, and IFN-γ levels were significantly decreased in severe H1N1 patients [30]. Further research is required to fully elucidate the mechanisms responsible for the differences in inflammatory responses between severe H7N9 and H5N1 patients.

Studies have indicated that monocyte HLA-DR recovery is a significant predictor of forthcoming sepsis [26]. In our study, the HLA-DR expression level on CD14^+^ cells exhibited an obvious negative correlation with the severity of H7N9 infection measured by the APACHE-II score of the patients. These findings suggest that HLA-DR expression in CD14^+^ cells is a potential biomarker for classifying severe and mild patients with H7N9 infection, or for predicting the disease progression of this disease.

In conclusion, our study provides a detailed immune characterization of patients with a novel avian influenza A (H7N9) virus infection, with a particular focus on the comparison of patients with severe and mild disease. In patients with severe disease, CD14^+^ cells were found to exhibit markedly reduced antigen-presenting capacity result in a reduced T cell response and decreased levels of cytokines in peripheral blood. In addition, this study indicate that expression of HLA-DR^+^ in CD14^+^ cells may be an potential biomarker for predicting the disease progression of H7N9 infection.

## Supporting Information

Figure S1 Leucocyte subset counts and their correlation with the severity of H7N9 infection. (A) The lowest values of leucocyte during disease progression versus APACHE-II score with Spearman's correlation coefficients. Each point represents an individual patient. The percentages of monocytes (B) and neutrophil (C) and the absolute numbers of neutrophil in patients during disease progression. Each line represents an individual patient. Results are shown for patients with severe H7N9 infection (red tones symbols and lines) with mild H7N9 infection (blue tone symbols and lines).
(TIF)

Figure S2 Levels of plasma cytokines and chemokines over the course of H7N9 infection. Plasma levels of TNF-α (A), IL-1β (B), IL-1Rα (C), IL-4 (D), IL-5 (E) and IL-6 (F) in patients with severe and mild H7N9 infection. Data represent mean ±SD. *, *p*<0.05. **, *p*<0.01, ***, *p*<0.001.
(TIF)

Figure S3 The percentages of lymphocyte subsets in peripheral blood from middle-aged and older health control. The distribution of CD3+ cells, CD4+ cells, CD8+ cells (A), NK cells, B cells (B), HLA-DR+ cells in PBMC, CD14+ cells and the percentages of HLA-DR expression on CD14+ monocytes (C) in middle-aged(50.77±1.88) and older(71.91±5.45) health control were detected by flowcytometry. Data represent mean ±SD.
(TIF)

Figure S4 Phagocytosis capacity of PBMC. (A) Phagocytic internalization of *E. coli* stained by DAPI (blue) in PBMC from severe and mild H7N9 patient was detected by fluorescence microscopy, Original magnification: ×400. (B) The percentages of *E. coli* ^+^ cells in *E. coli* stimulated PBMC from young healthy control (aged 30 to 35 years, n = 4) and old healthy control (aged 60 to 65 years, n = 4), Data represent mean ±SD.
(TIF)

Figure S5 In vitro analysis of cytokine and chemokine production by patients PBMC with polyI:C stimulation. The levels of cytokines IL-12 (A), IFN-γ (B), TNF-α (C), IL-4 (D), IL-10 (E), IL-17A (F) and chemokines IP-10 (G), MCP-1 (H), RANTES (I) secreted by polyI:C (20 ng/ml) stimulated PBMC from patients with severe and mild H7N9 infection. Data represent mean ± SD.
(TIF)
