# Supplementary figures and images for: Severe H7N9 Infection Is Associated with Decreased Antigen-Presenting Capacity of CD14+ Cells
Source: PLoS One. 2014 Mar 24;9(3):e92823. doi: 10.1371/journal.pone.0092823 (PMC3963940; doi:10.1371/journal.pone.0092823)

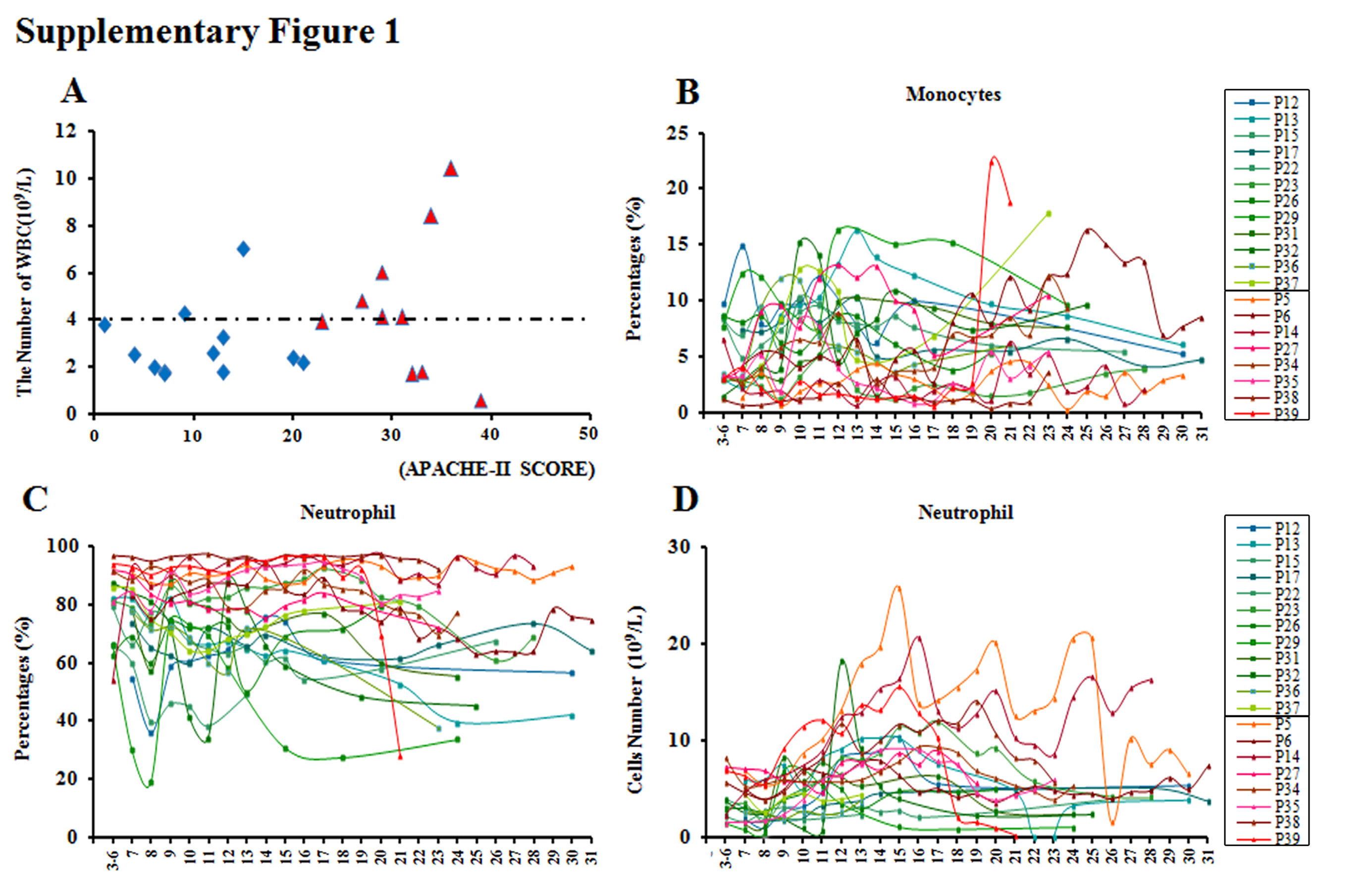

Supplement: Figure S1 — Leucocyte subset counts and their correlation with the severity of H7N9 infection. (A) The lowest values of leucocyte during disease progression versus APACHE-II score with Spearman's correlation coefficients. Each point represents an individual patient. The percentages of monocytes (B) and neutrophil (C) and the absolute numbers of neutrophil in patients during disease progression. Each line represents an individual patient. Results are shown for patients with severe H7N9 infection (red tones symbols and lines) with mild H7N9 infection (blue tone symbols and lines). (TIF) [file pone.0092823.s001.tif]

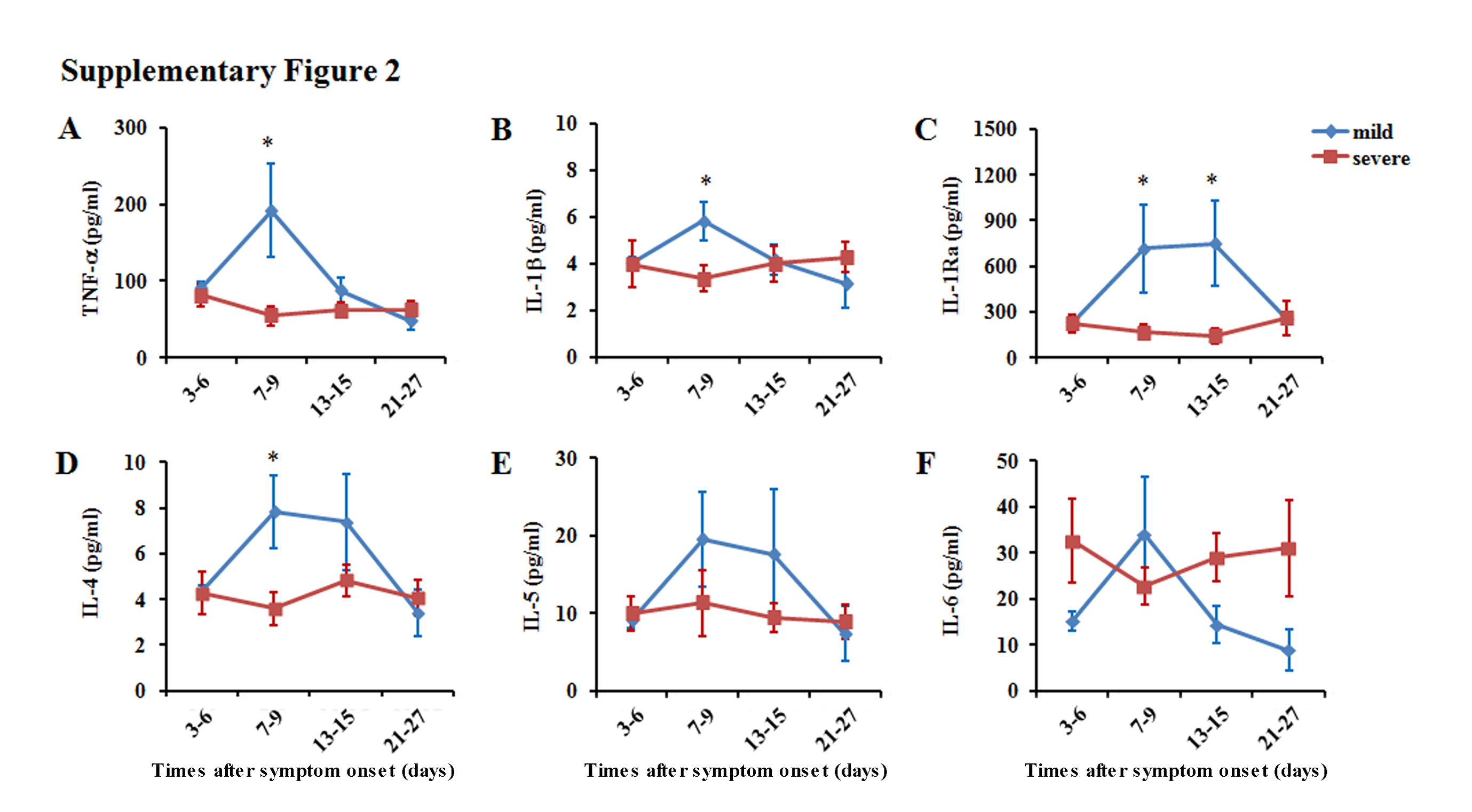

Supplement: Figure S2 — Levels of plasma cytokines and chemokines over the course of H7N9 infection. Plasma levels of TNF-α (A), IL-1β (B), IL-1Rα (C), IL-4 (D), IL-5 (E) and IL-6 (F) in patients with severe and mild H7N9 infection. Data represent mean ±SD. *, p<0.05. **, p<0.01, ***, p<0.001. (TIF) [file pone.0092823.s002.tif]

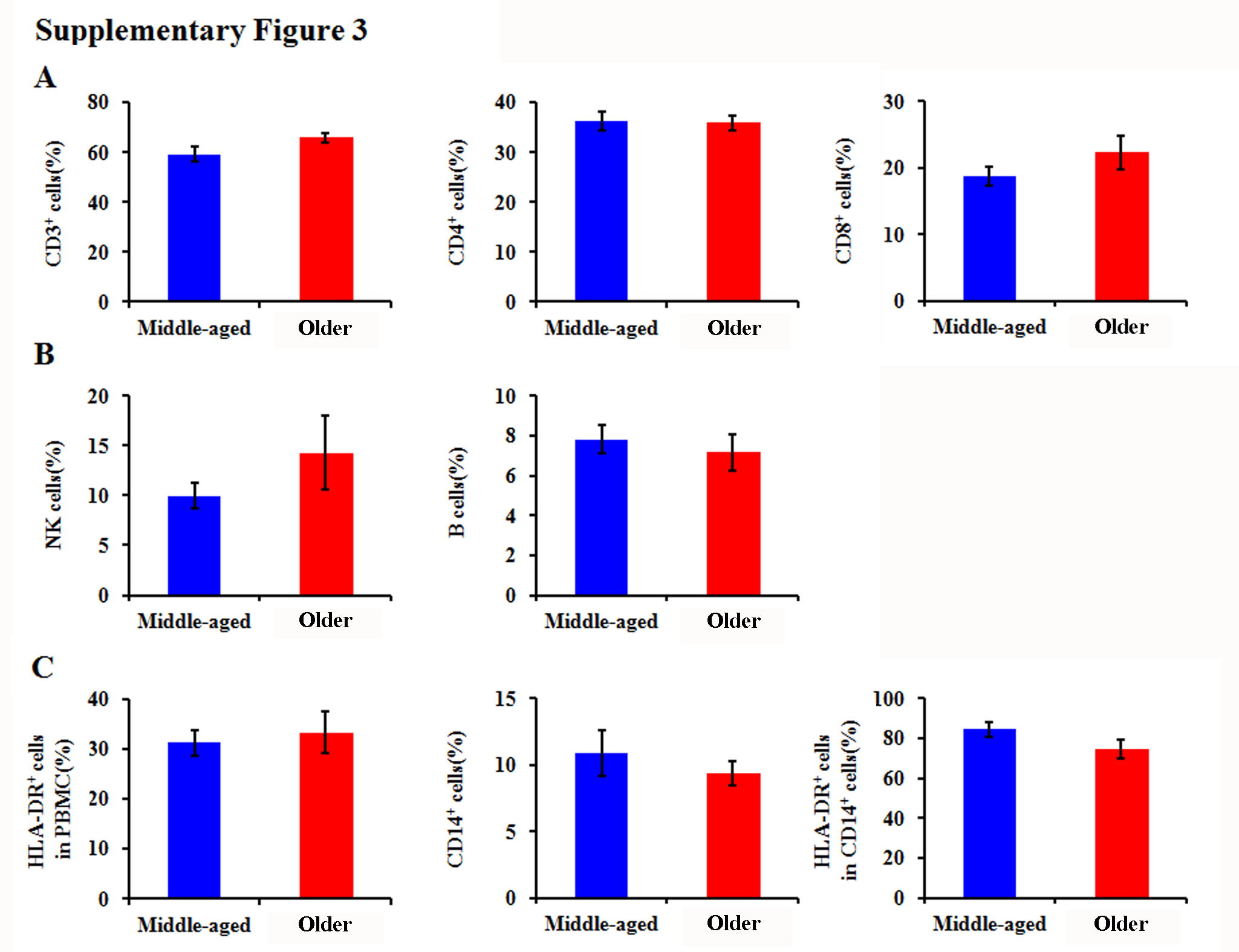

Supplement: Figure S3 — The percentages of lymphocyte subsets in peripheral blood from middle-aged and older health control. The distribution of CD3+ cells, CD4+ cells, CD8+ cells (A), NK cells, B cells (B), HLA-DR+ cells in PBMC, CD14+ cells and the percentages of HLA-DR expression on CD14+ monocytes (C) in middle-aged(50.77±1.88) and older(71.91±5.45) health control were detected by flowcytometry. Data represent mean ±SD. (TIF) [file pone.0092823.s003.tif]

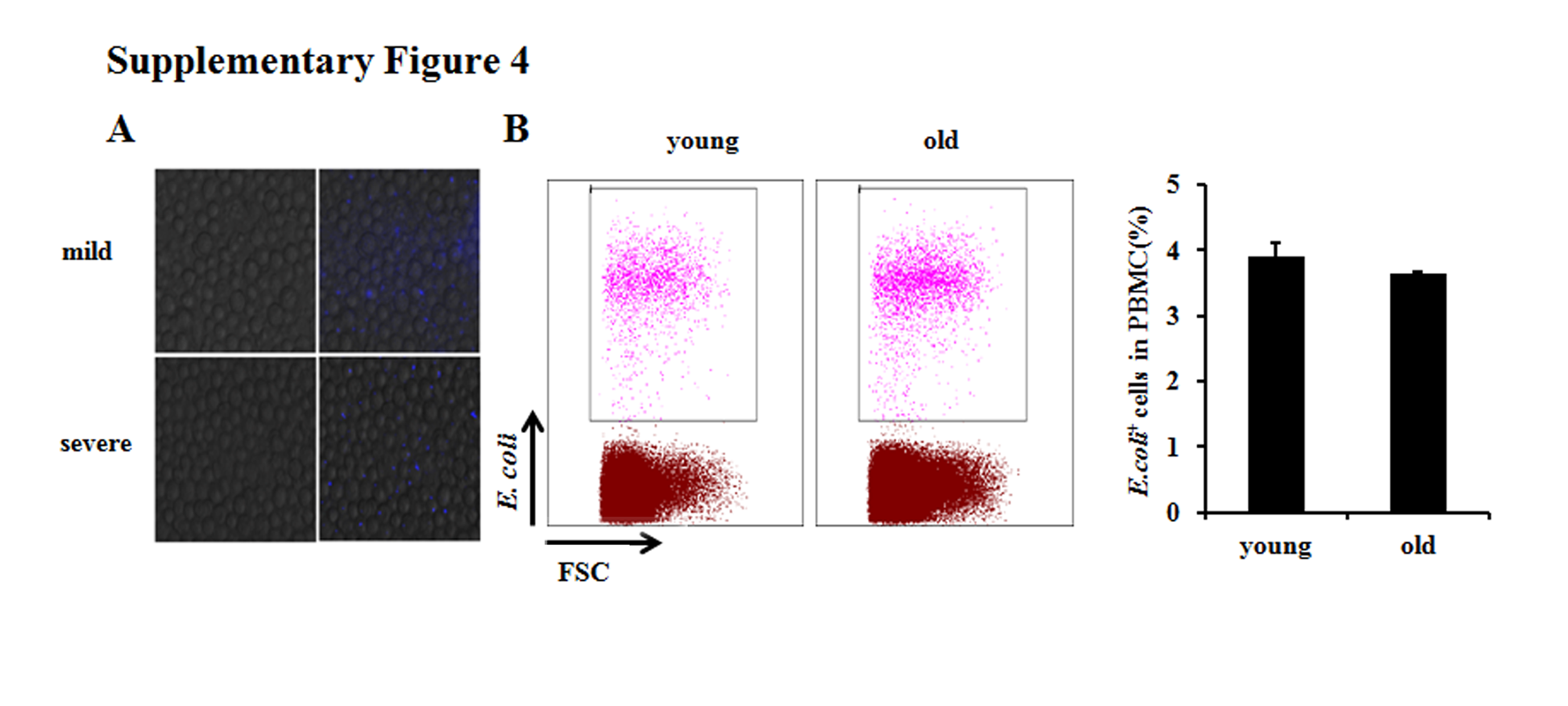

Supplement: Figure S4 — Phagocytosis capacity of PBMC. (A) Phagocytic internalization of E. coli stained by DAPI (blue) in PBMC from severe and mild H7N9 patient was detected by fluorescence microscopy, Original magnification: ×400. (B) The percentages of E. coli + cells in E. coli stimulated PBMC from young healthy control (aged 30 to 35 years, n = 4) and old healthy control (aged 60 to 65 years, n = 4), Data represent mean ±SD. (TIF) [file pone.0092823.s004.tif]

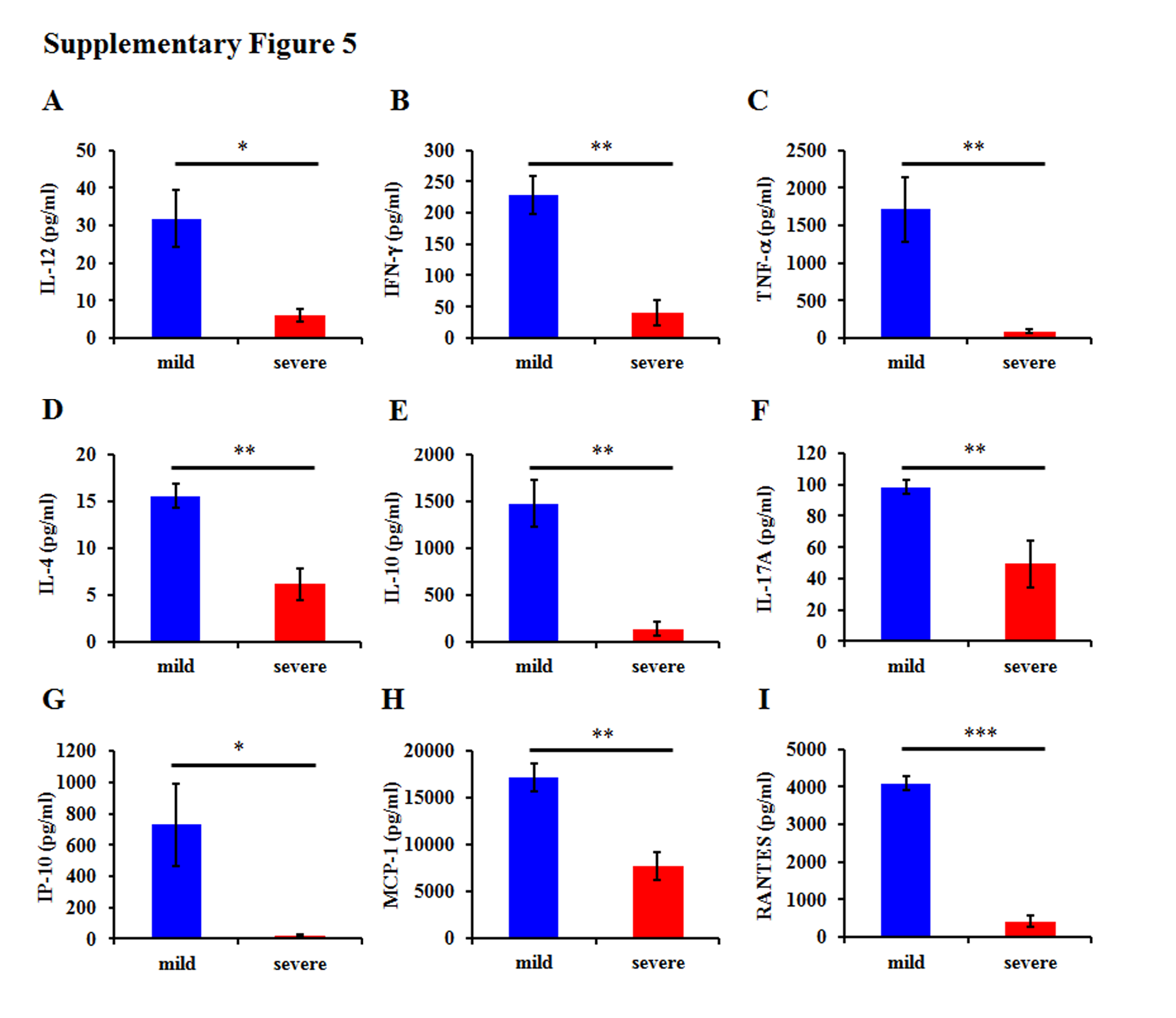

Supplement: Figure S5 — In vitro analysis of cytokine and chemokine production by patients PBMC with polyI:C stimulation. The levels of cytokines IL-12 (A), IFN-γ (B), TNF-α (C), IL-4 (D), IL-10 (E), IL-17A (F) and chemokines IP-10 (G), MCP-1 (H), RANTES (I) secreted by polyI:C (20 ng/ml) stimulated PBMC from patients with severe and mild H7N9 infection. Data represent mean ± SD. (TIF) [file pone.0092823.s005.tif]
